# Toll-Like Receptor 4 Prompts Human Breast Cancer Cells Invasiveness via Lipopolysaccharide Stimulation and Is Overexpressed in Patients with Lymph Node Metastasis

**DOI:** 10.1371/journal.pone.0109980

**Published:** 2014-10-09

**Authors:** Huan Yang, Bo Wang, Tao Wang, Longjiang Xu, Chunyan He, Huiyan Wen, Jie Yan, Honghong Su, Xueming Zhu

**Affiliations:** 1 Department of Clinical Laboratory, The Second affiliated Hospital of Soochow University, Suzhou, Jiangsu, China; 2 Department of Oncology, The Second affiliated Hospital of Soochow University, Suzhou, Jiangsu, China; 3 Department of Pathology, The Second affiliated Hospital of Soochow University, Suzhou, Jiangsu, China; University of Kansas Medical Center, United States of America

## Abstract

Toll-like receptor (TLR)4-mediated signaling has been implicated in tumor cell invasion, survival, and metastasis in a variety of cancers. This study investigated the expression and biological role of TLR4 in human breast cancer metastasis. MCF-7 and MDA-MB-231 are human breast cancer cell lines with low and high metastatic potential, respectively. Using lipopolysaccharide (LPS) to stimulate MCF-7 and MDA-MB-231 cells, expression of TLR4 mRNA and protein increased compared with that in control cells. TLR4 activation notably up-regulated expression of matrix metalloproteinase (MMP)-2, MMP-9 and vascular endothelial growth factor(VEGF) mRNA and their secretion in the supernatants of both cell lines. LPS enhanced invasion of MDA-MB-231 cells by transwell assay and MCF-7 cells by wound healing assay. LPS triggered increased expression of TLR4 downstream signaling pathway protein myeloid differentiation factor 88(MyD88) and resulted in interleukin (IL)-6 and IL-10 higher production by human breast cancer cells. Stimulation of TLR4 with LPS promoted tumorigenesis and formed metastatic lesions in liver of nude mice. Moreover, expression of TLR4 and MyD88 as well as invasiveness and migration of the cells could be blocked by TLR4 antagonist. Combined with clinicopathological parameters, TLR4 was overexpressed in human breast cancer tissue and correlated with lymph node metastasis. These findings indicated that TLR4 may participate in the progression and metastasis of human breast cancer and provide a new therapeutic target.

## Introduction

Despite advances in treatment of breast cancer, the effective control of metastasis remains a complex problem. It was reported that over 90% of the deaths of cancer patients is caused by metastasis, which is formed by the spread of disseminated primary tumor cells to distant anatomic sites [1]. In Asia, the age-adjusted mortality rate is 9.5 deaths per 100 000, and in China, it is 3.4–5.7 deaths per 100 000 [2]. Finding new modalities that treat the local and systemic components of the disease, in particular for patients who do not respond to conventional treatments, has become increasingly important.

Toll-like receptors (TLRs) are members of the interleukin-1 receptor (IL-1R) superfamily that share significant homology in their cytoplasmic regions, the Toll/IL-1R (TIR) domain [3], [4]. TLRs play a crucial role in the inflammation and innate host defense against invading microorganisms by recognizing conserved motifs of microbial origin, also known as pathogen-associated molecular patterns (PAMPs) [5], [6]. Evidence suggests that several PAMPs can stimulate TLR4. These molecules include LPS from Gram-negative bacteria, fusion protein from respiratory syncytial virus, and envelope protein from mouse mammary tumor virus [7], [8]. In addition to microbial ligands, several endogenous ligands have been reported to stimulate TLRs. These include the heat-shock 60-kDa protein (HSP60) and HSP70, oligosaccharides of hyaluronan [9], and high-mobility group box 1 (HMGB1) [10].

Toll-like receptors (TLRs) have garnered an extraordinary amount of interest in cancer research due to their role in tumor progression. TLR4 has already been linked to tumors such as ovarian, prostate and head and neck cancers [11]–[13]. However, little research has investigated the role of TLR4 in breast cancer progression [14], [15], and there were discrepancies of TLR4 among these studies, the mechanism of either antitumor or tumor-promoting activities is unknown. For instance, TLR4 activation on metastatic breast cancer cells promoted the αvβ3-mediated adhesion and invasiveness of cancer cells [15]. While in particular, silencing of TLR4 promotes tumor progression and lung metastasis which exerts a negative role at the cancer cell level in a murine metastatic breast tumor model [16].

Our previous studies have found that TLR4 expressed higher levels than any other TLRs and knockdown of TLR4 could actively inhibit proliferation and survival of human breast cancer cells MDA-MB-231 [17]. In the present study, we aimed to investigate the function of TLR4/MyD88 signaling in tumor progression in both cell lines MDA-MB-231 and MCF-7, especially in tumor metastasis. We also explored TLR4 expression in breast cancer tissue and the relation between TLR4 expression and tumor metastasis. The results may provide further insight to a strategy for breast cancer therapy.

## Materials and Methods

### Chemicals and reagents

LPS (from *Escherichia coli* 0111:B4; Sigma, St Louis, MO, USA) was stored in a stock solution of 1 mg/ml at −20°C, and was diluted to various concentrations with serum-free culture medium when used. TLR4 antagonist eritoran was provided by Eisai, Inc., USA. ELISA kits for MMP-2, MMP-9, and VEGF were obtained from RayBiotech (Norcross, GA, USA). Synthesis of primers were made by Sangon Biotechnology (Shanghai, China).

### Cell culture and Treatment

Human breast cancer cell lines MDA-MB-231 and MCF-7 were purchased from the Type Culture Collection of the Chinese Academy of Sciences (Shanghai, China). MDA-MB-231 cells were cultured in L-15 medium (Gibco, USA) medium supplied with 10% fetal bovine serum (FBS, Gibco), 100 µg/ml streptomycin, 100 IU/ml penicillin in normal air atmosphere at 37°C. MCF-7 cells were cultured in Dulbecco's Modified Eagle's Medium (DMEM) (Gibco) supplemented with 10% FBS, 100 µg/ml streptomycin, 100 IU/ml penicillin in 5% CO2 atmosphere at 37°C.

The experiment was designed in four groups: Control, LPS: for LPS treatment, MCF-7 and MDA-MB-231 were treated with LPS at various concentrations (0, 2, 10 and 20 µg/ml). Eritoran (ER): MCF-7 and MDA-MB-231 were pretreated with 100 nmol/L of eritoran for 30 min. ER+LPS: for TLR4 blocking, 100 nmol/L of eritoran was added to the cell culture for 30 min prior to the addition of LPS.

### Reverse transcriptase polymerase chain reaction (RT-PCR)

Total RNA was extracted using Trizol reagent (Invitrogen, Carlsbad, CA, USA), and the concentration of RNA was determined by absorbance at 260 nm in relation to absorbance at 280 nm. Two micrograms of total RNA was reverse transcribed to cDNA by Moloney murine leukemia virus reverse transcriptase (Thermo, USA). The 50-µl reaction mixture contained 19 µl nuclease-free water, 2.0 µl cDNA, 2.0 µl (10 µmol/l) each primer, and 25 µl PCR Master Mix (2×) (Thermo). The primer sequences are listed in Table S1. The amplification cycle started with denaturation at 94°C for 5 min, followed by 35 cycles of denaturation at 94°C for 30 s, annealing (GAPDH at 55°C, TLR4 at 58°C, VEGF at 57°C, MMP-9 at 61°C, MMP-2 at 57°C) for 30 s, and 72°C for 10 min. PCR products were analyzed on 1–2% (wt/vol) agarose gels containing 0. 5 µg/ml ethidium bromide and were visualized under UV light. The ratio of TLR4 to GAPDH served as the level of mRNA expression.

### Real-time PCR

Real-time PCR was performed to detect TLR4, MMP-2, MMP-9 and VEGF gene expression. The 20-µl reaction mixture contained 7.0 µl nuclease-free water, 1.0 µl cDNA (1 µg/µl), 1.0 µl (10 µM) each primer and 10.0 µl Maxima SYBR Green/ROX qPCR Master Mix (2×) (Thermo). The thermal cycle profile for PCR was as follows: 94°C for 5 min, 40 cycles of PCR (94°C for 30 s; (GAPDH at 55°C, TLR4 at 58°C, VEGF at 57°C, MMP-9 at 61°C, MMP-2 at 57°C) for 30 s; 72°C for 30 s). The PCR program was measured with CFX Connect Real-Time PCR Detection System (Bio-Rad, USA). The 2^−ΔΔCT^ method [18] was used to analyze the relative expression of each gene.

### Flow cytometry

The expression of TLR4 was measured by flow cytometry: MCF-7 and MDA-MB-231 (5×10^5^) cells were treated with 2 µg/ml LPS for 48 h. Cells were collected and washed three times with PBS followed by incubation with 3 µl purified rabbit anti-humanTLR4 antibody (Santa Cruz Biotechnology, Santa Cruz, CA, USA) for 30 min. Subsequently, cells were washed three times with PBS and incubated with 2 µl phycoerythrin-labeled goat anti-rabbit IgG mAb (R&D, Minneapolis, MN, USA) for 30 min away from light. Cells were washed again three times in PBS. Afterwards the cells were analyzed for expression of TLR4 using flow cytometry [FACScalibur; Becton Dickinson (BD), Franklin Lakes, NJ, USA], and the data were processed with BD Cell-Quest software. The negative control was performed by omitting the anti TLR4 antibody. The expression of cytokines was measured by flow cytometry: MCF-7 and MDA-MB-231 cells were treated with various concentrations of LPS (0, 2, 10 and 20 µg/ml) for 48 h, the culture supernatants were harvested, and the cytokines were measured by CBA human Th1/Th2 cytokine kit (BD) according to the manufacturer's instructions. The data were obtained from flow cytometry and processed with BD Cell-Quest software.

### Invasion assay

Matrigel (BD) was diluted (1∶5) in serum-free DMEM, and the mixture (50 µl) was added to each chamber at low temperature (on ice) and maintained at 37°C for 30 min. After Matrigel was polymerized sufficiently, 600 µl DMEM containing 10% FBS was added to the bottom of the chambers and neutralized for 1 h. The LPS group was added with 10 µg/ml LPS. The blocking(ER+LPS) group, 100 nmol/L eritoran was added to the cell culture for 30 min prior to the addition of 10 µg/ml LPS. Cell suspension (5×10^5^) was seeded into the upper chambers. After culture for 48 h, the cells were fixed with 2% paraformaldehyde in 1× PBS and stained with 0.1% crystal violet for 30 min. After wiping off the inside of the chambers using cotton-tipped applicators, invasive cells remained on the bottom of the membranes and total number of the cells through the matrigel was counted under the microscope in the whole fields.

### Wound healing assay

MCF-7 cells were removed by trypsinization, counted, and plated at 1×10^6^ cells/ml in 12-well dishes. The LPS group was added with 10 µg/ml LPS. The blocking(ER+LPS) group, 100 nmol/L eritoran was added to the cell culture for 30 min prior to the addition of 10 µg/ml LPS. Cells were incubated for yielding a confluent cell layer for wounding. Wounds were made using a pipette tip and photographs taken immediately (time 0), 24 and 48 h after wounding. Experiments were carried out in triplicate and repeated at least three times.

### ELISA

Cells were seeded on six-well plates at a density of 2×10^6^/well. Cells were treated with experimental LPS concentrations (0, 2, 10 and 20 µg/ml) for 48 h. MMP-2, MMP-9 and VEGF in the supernatants were measured by ELISA kit (R&D, Minneapolis, MN, USA)according to the manufacturer's instructions. The absorbance values were read within 30 min using an automatic microplate spectrophotometer (340 st; Anthos Zenyth, Salzburg, Austria) at 450 nm (A_450_). The average A450 values were calculated for each set of reference standards, controls, and samples. A standard curve was constructed by plotting the mean absorbance obtained for each reference standard against its concentration using Excel software. Using the mean absorbance value for each sample, the corresponding concentration was determined from the standard curve.

### Western blotting

MCF-7 and MDA-MB-231 cells in LPS group were treated with 2 µg/ml LPS for 48 h. In TLR4 blocking group, 100 nmol/L eritoran was added to the cell culture for 30 min prior to the addition of 2 µg/ml LPS for 48 h. For western blot analysis, 1×10^6^ cells were lysed in modified RIPA buffer (Beyotime, Shanghai, China). The protein concentration was measured by BCA Protein Assay Kit (Beyotime) and samples were loaded on 10% SDS-PAGE. Following electrophoresis, the proteins were transferred to a BioTrace PVDF membrane (Pall Life Sciences, Ann Arbor, MI, USA) and blocked in 5% non-fat dried milk for 1 h at room temperature and incubated with rabbit antibodies against TLR4 (1∶1000; Abcam, Cambridge, MA, USA), MyD88 (1∶500, Boster, Wuhan, China) and GAPDH (1∶5000, Abcam, Cambridge, MA, USA) overnight at 4°C. After washing three times with Tris-buffered saline Tween 20 (TBST), membranes were incubated with anti-rat IgG (1∶5000, Bioworld, Dublin, OH, USA). The membrane was washed three times with TBST, and signals were detected by enhanced chemiluminescence system (Amersham Pharmacia Biotech, Bucks, UK).

### Animal model

All animal work was conducted according to relevant national and international guidelines. In accordance with the recommendations of the Weatherall report. Animals were maintained in accordance with institutional policies, and all studies were performed with approval of the Committee on Use and Care of Animals of The Second Affiliated Hospital of Soochow University. Female BALB/c nu/nu mice aged 4–6 weeks were obtained from Laboratory Animal Center of Shanghai, Academy of Science, China. After all mice were given an subcutaneous injection of 100 µl anesthetic solution containing 15% narketan and 15% xylapan, tumor cells were subcutaneously injected in the right flank. The MDA-MB-231 tumor model, mice (n = 5) in the control group were inoculated with a suspension of 1×10^7^ cells in 30 µl PBS. Mice (n = 5) in the LPS group were inoculated with a suspension of 1×10^7^ cells cultured with 10 µg/ml LPS for 48 h. After tumor cell inoculation, the primary flank tumor volume was measured. Tumor volumes were assessed using calipers. Tumor volume was calculated as V = L×W^2^/2, where L is the length and W the width of the tumor. Mice in the MDA-MB-231 model were fed on experimental diets for 24 days. Then tumors, lungs and livers were excised, weighed and stored in 10% formalin solution for at least 24 h, after which, they were embedded in paraffin wax.

### Patients

Primary tumor samples and normal breast tissue samples were obtained from The Second Affiliated Hospital of Soochow University (Suzhou, China) (from August 2012 to June 2013). Informed consent was given in all donators examined and the informed consent was written. All experiment protocols were approved by the Ethics Committee of The Second Affiliated Hospital of Soochow University. All investigation was conducted according to the principles expressed in the Declaration of Helsinki. Normal breast tissue samples were obtained from 10 patients undergoing surgery for non-neoplastic and noninflammatory disorders. Twenty-two Patients participating in the study did not receive radiotherapy, chemotherapy or biological therapy. For mRNA analysis, some fresh specimens were frozen in liquid nitrogen immediately after excision and then marked and stored at −80°C for RT-PCR and real-time RT-PCR analysis (as described previously). For immunohistochemical analysis, some fresh specimens were marked and stored in formaldehyde (4%), while another part of the tumor was transferred to the pathology laboratory for confirmation of breast cancer diagnosis. Routine pathological variables including age, tumor grading, tumor type and immunohistochemical determination of progesterone receptor and estrogen receptor status are illustrated in Table S2.

### Immunohistochemical analysis

Formalin-fixed, paraffin-embedded tissue sections (4 µm) were deparaffinized in xylene, rehydrated through a series of ethanol dilutions, and boiled for 10 min in citrate buffer (10 mM, pH 6.0). Endogenous peroxidase activity was suppressed by exposure to 3% hydrogen peroxide for 10 min. Slides were blocked with 5% bovine serum albumin (Boster, Wuhan, China) and incubated with anti-TLR4 polyclonal antibody (1∶50; Abcam) for 1 h at 37°C. A goat anti-rabbit secondary antibody was used according to the protocol supplied with the ABC Staining System kit (Boster). Sections probed with PBS as primary antibody were considered negative controls. For digital image analysis, the software Adobe Photoshop version 6.0 was used. Results were scored by two independent pathologists as positive, heterogeneous, or negative, when the percentage of stained tumor cells in each section was >75%, between 25% and 75%, and <25%, respectively. The two scores were averaged. The level of staining intensity was recorded as none, weak, moderate, or strong.

### Statistical analysis

GraphPad Prism software (San Diego, CA, USA) was used to perform statistical comparisons between different values. Data were expressed as the means ± standard deviation (SD). Statistical significance was determined by Student's *t* test and one way ANOVA, and differences were considered significant at P<0.05.

## Results

### Expression of TLR4 in human breast cancer cells and association of TLR4 up-regulation with LPS stimulation

**Figure 1:**
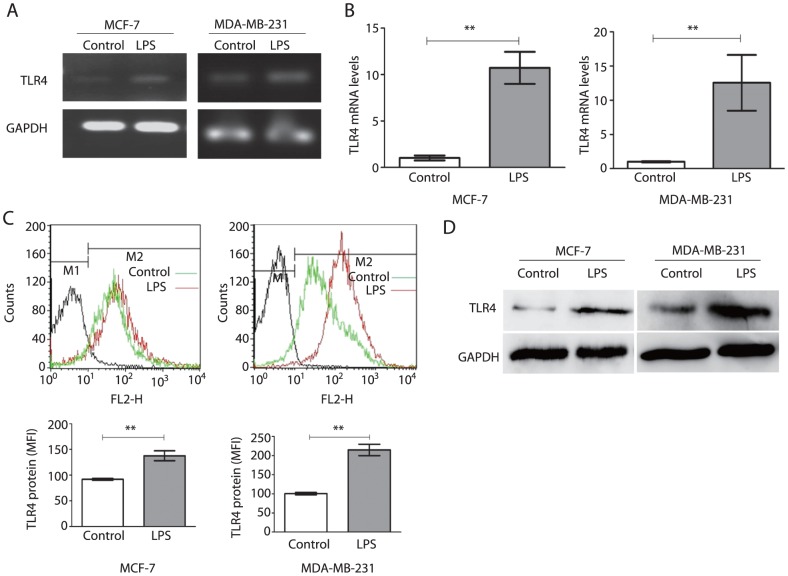
Expression of TLR4 in human breast cancer cells. (A) Expression of TLR4 in control and stimulated (2 µg/ml LPS) MCF-7 and MDA-MB-231 cells for 48 h by RT-PCR. (B) Expression of TLR4 in control and LPS groups(2 µg/ml, 48 h) was analyzed by real-time PCR, **P<0.05. Each bar represented triplicate analyses of mean±SD. (C) Expression of TLR4 in control and LPS groups (2 µg/ml, 48 h)was analyzed by flow cytometry,**P<0.05. Each bar represented triplicate analyses of mean±SD. (D) Expression of TLR4 in control and LPS groups(2 µg/ml, 48 h) was analyzed by western blotting. All results were representative of three separate experiments.

Expression of TLR4 on MCF-7 and MDA-MB-231 human breast cancer cell lines was analyzed by RT-PCR, real time-PCR, flow cytometry and western blotting. Both of the breast cancer cell lines expressed TLR4 mRNA detected by RT-PCR (Fig. 1A) and protein was detected by western blotting (Fig. 1D), and after LPS stimulation, the increased expression of TLR4 was clearly observed. Quantitative analysis of the increased expression of TLR4 mRNA and protein by real time-PCR and flow cytometry showed that, at the mRNA level, TLR4 expression in MCF-7 cells in the stimulated group was 10.51±1.73 times higher than in the controls (P<0.05), and it was 12.56±4.07 times higher in MDA-MB-231 cells (P<0.05), as shown in Fig. 1B. Similarly, TLR4 was significantly upregulated at the protein level (P<0.05), as shown in Fig. 1C.

### LPS up-regulated MMP-2, MMP-9 and VEGF production by human breast cancer cells

**Figure 2:**
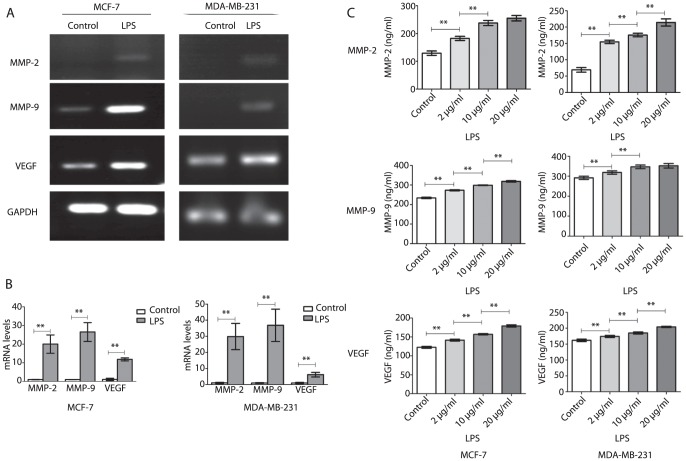
LPS upregulated MMP-2, MMP-9 and VEGF production by MCF-7 and MDA-MB-231 cells. (A) mRNA of MMP-2, MMP-9 and VEGF were analyzed by RT-PCR in control and LPS (2 µg/ml, 48 h) groups. (B) mRNA of MMP-2, MMP-9 and VEGF were analyzed by real-time PCR in control and LPS groups(2 µg/ml, 48 h). Each bar represented triplicate analyses of mean±SD, **P<0.05. (C) MMP-2, MMP-9 and VEGF in the culture supernatants of control and LPS groups (0, 2, 10 and 20 µg/ml) for 48 h was measured by ELISA. Each bar represented triplicate analyses of mean±SD, **P<0.05.

To investigate cell invasive capacity, genes related to angiogenesis and invasion were studied in MCF-7 and MDA-MB-231 cells. MMP-2, MMP-9 and VEGF expression was obviously increased in both MCF-7 and MDA-MB-231 cells treated with LPS at the mRNA level, as detected by RT-PCR (Fig. 2A). For further quantitative analysis, the real-time PCR results showed that, after treatment with LPS, MMP-2, MMP-9 and VEGF mRNA expression in MCF-7 cells was 20.0±4.92 (P<0.05), 26.48±5.03 (P<0.05) and 11.84±0.80 (P<0.05) times higher, respectively, than in the controls. Similarly, MMP-2, MMP-9 and VEGF mRNA expression in MDA-MB-231 cells was 29.84±8.17 (P<0.05), 36.85±10.04 (P<0.05) and 6.22±1.44 (P<0.05) times higher, respectively, than in the controls (Fig. 2B). At the protein level, with increasing concentration of LPS, the production of MMP-2, MMP-9 and VEGF in the culture supernatants of MCF-7 cells was significantly increased (P<0.05), except that, MMP-2 was not significantly changed by treatment with 10 and 20 µg/ml LPS (P>0.05) (Fig. 2C). The result with MDA-MB-231 cells was similar, and the production of MMP-9 also had no significant change when treated with LPS at a concentration of 10 and 20 µg/ml (P>0.05) (Fig. 2C).

### Stimulation of TLR4 with LPS enhanced invasion of MDA-MB-231 and wound healing of MCF-7 cells

**Figure 3:**
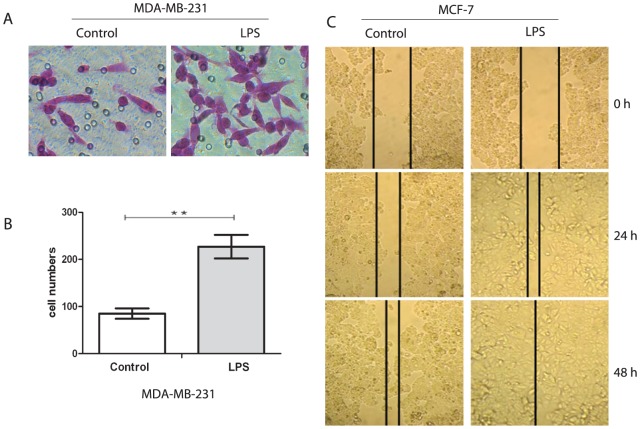
Stimulation of TLR4 promoted migration and invasion of breast cancer cell lines. (A) Matrigel invasion assay was performed to observe the changes in MDA-MB-231 cell invasion. The LPS group was stimulated with 10 µg/ml LPS for 48 h. Magnification, ×200. (B) Total number of invading cells through the matrigel was counted under the microscope in the whole fields. Each bar represents the mean±SD of three wells counted. **P<0.05. Results are representative of three separate experiments. (C) Wound-healing assay was performed to observe the changes in MCF-7 cell invasion after stimulation. The LPS group was also stimulated with 10 µg/ml LPS for 48 h. Magnification, ×100. All results were representative of three separate experiments.

One of the key functions of cancer cells is promotion of invasion; a process crucial for tumor metastasis. To evaluate the impact of TLR4 on invasion of MCF-7 and MDA-MB-231 human breast cancer cells, a matrigel invasion assay using LPS-treated cells was performed. The functional significance of TLR4 expression was suggested by the ability of MDA-MB-231 cells to migrate toward TLR4. The number of MDA-MB-231 cells that migrated in response to TLR4 stimulated with LPS was significantly higher than that for unexposed cell lines (Fig. 3A). The number of migrated cells was 227±19 in the stimulated group, which was clearly higher than that in the controls with 85±8 (P<0.05) (Fig. 3B). However, MCF-7 cells whether treated with LPS or not failed to migrate through the transwells. The wound-healing assay was used to assess the impact of TLR4 stimulated with LPS on invasion of MCF-7 cells. As shown in Fig. 3C, the stimulated group was basically healed at 24 h, which was clearly faster than in the control group.

### LPS triggered increased expression of MyD88, IL-6 and IL-10 by human breast cancer cells

**Figure 4:**
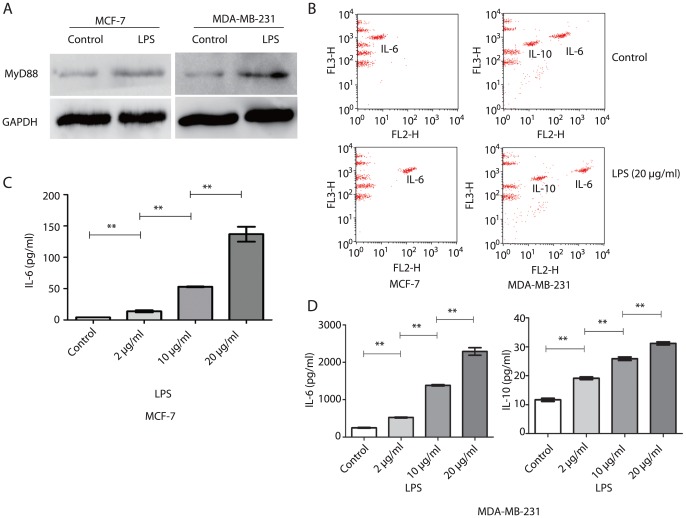
LPS promoted MyD88, IL-6 and IL-10 production by human breast cancer cells. (A) Expression of MyD88 was analyzed by western blotting. LPS stimulation (2 µg/ml) for 48 h induced higher expression of MyD88 in both cell lines. (B) Representative images of IL-6 and IL-10 in supernatants from control and LPS groups (20 µg/ml, 48 h) of both cell lines measured by flow cytometry. (C) and (D) Quantitative analysis of cytokines in supernatants of MCF-7 and MDA-MB-231 cells treated with LPS (0, 2, 10 and 20 µg/ml) for 48 h. Each bar represents triplicate analyses of mean±SD, **P<0.05. All results were representative of three separate experiments.

TLR4 signaling has been divided into MyD88-dependent and MyD88-independent pathways. Based on studies that MyD88-dependent pathway was shown to be responsible for proinflammatory cytokine expression, while the MyD88-independent pathway mediates the induction of Type I interferons and interferon-inducible genes. We measured the production of MyD88 and cytokine expression capacity of human breast cancer response to LPS. Western blotting showed that, after treatment with LPS, MyD88 expression was higher in MCF-7 and MDA-MB-231 cells than the controls (Fig. 4A). Cytokine expression measured by flow cytometry was shown in Fig. 4B. With increasing concentration of LPS, the production of IL-6 in the culture supernatants of MCF-7 cells was significantly increased (P<0.05) (Fig. 4C), and the production of IL-6 and IL-10 in the culture supernatants of MDA-MB-231 cells was also significantly increased (P<0.05), as shown in Fig. 4D. However, the production of IL-10 in the culture supernatants of MCF-7 cells was not detected.

### Stimulation of TLR4 with LPS promoted tumorigenesis in nude mice

**Figure 5:**
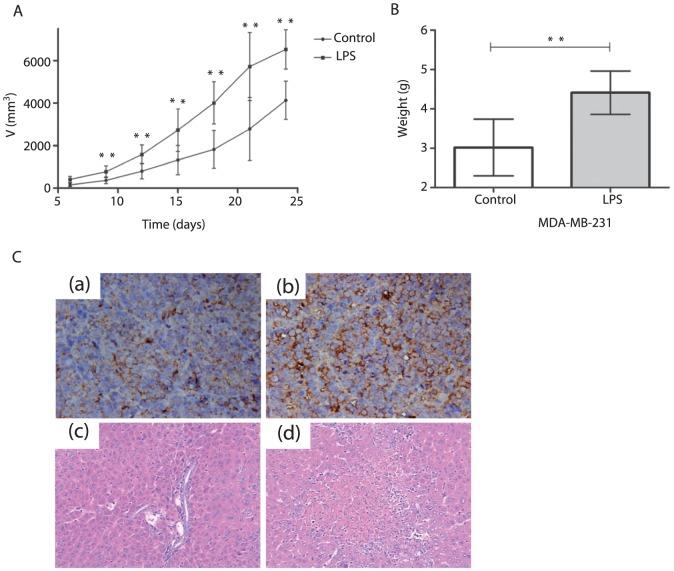
TLR4 stimulation with LPS promoted tumorigenesis in the tumor model of MDA-MB-231. (A) Comparison of breast tumor volume from LPS treatment group. The mean±SD tumor volume of five animals per group. **P<0.05. (B) Comparison of breast tumor weight from LPS treatment group. Each bar represents the mean±SD tumor weight of five animals per group. **P<0.05. (C) TLR4 expression in transplanted tumor sections. Magnification,×200. (a) and (b) Immunohistochemistry for TLR4 in transplanted tumor sections. (a) is control group and (b) is LPS group. (c) H&E staining of the liver from control group. (d) H&E staining of the liver from LPS group. Magnification,×100.

The transplanted tumor formation rate in the control and LPS groups of MDA-MB-231 cells was 100% (5/5). Eighty percent (4/5) of the transplanted tumors in the LPS group showed necrosis and ulceration, compared with 40% (2/5) in the control group. A marked increase in tumor volume was observed in mice in the LPS group (Fig. 5A). In addition, when the mice were sacrificed, the tumor weight in LPS group was higher than in the control group (Fig. 5B). Immunohistochemistry confirmed that LPS-treated MDA-MB-231 tumor had a significantly increased level of TLR4 protein expression (Fig. 5C (a)(b)). Hematoxylin and eosin (H&E) staining confirmed that microscopic invasive metastases were found in the livers of the LPS group (Fig. 5C(c)(d)). Metastatic lesions in the liver were observed in 40% (2/5) of mice in the LPS group, and not found in other mice of control group. Microscopic metastases were not found in the lungs.

### Invasiveness and migration in breast cancer cells could be blocked by TLR4 antagonist

**Figure 6:**
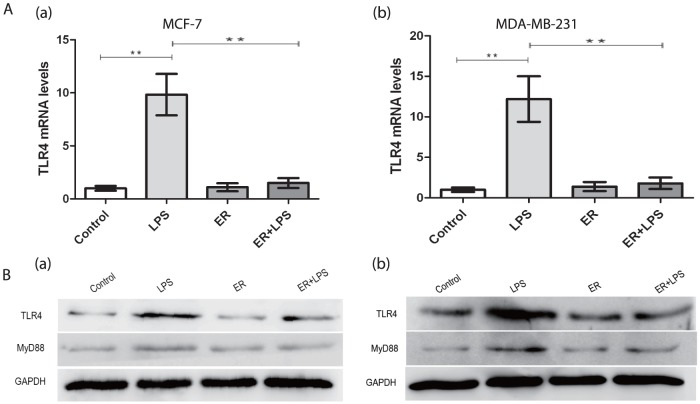
Effects of TLR4 antagonist eritoran on LPS-induced expression of TLR4 and MyD88 in human breast cancer cells. (A)Expression of TLR4 was analyzed by real-time PCR. **P<0.05. Each bar represented triplicate analyses of mean±SD. (B)Expression of TLR4, MyD88 was analyzed by western blotting. All results were representative of three separate experiments. LPS (2 µg/ml, 48 h), ER(100 nmol/L eritoran, 30 min pretreatment, 48 h), ER+LPS(100 nmol/L eritoran, 30 min prior to 2 µg/ml LPS, 48 h).

**Figure 7:**
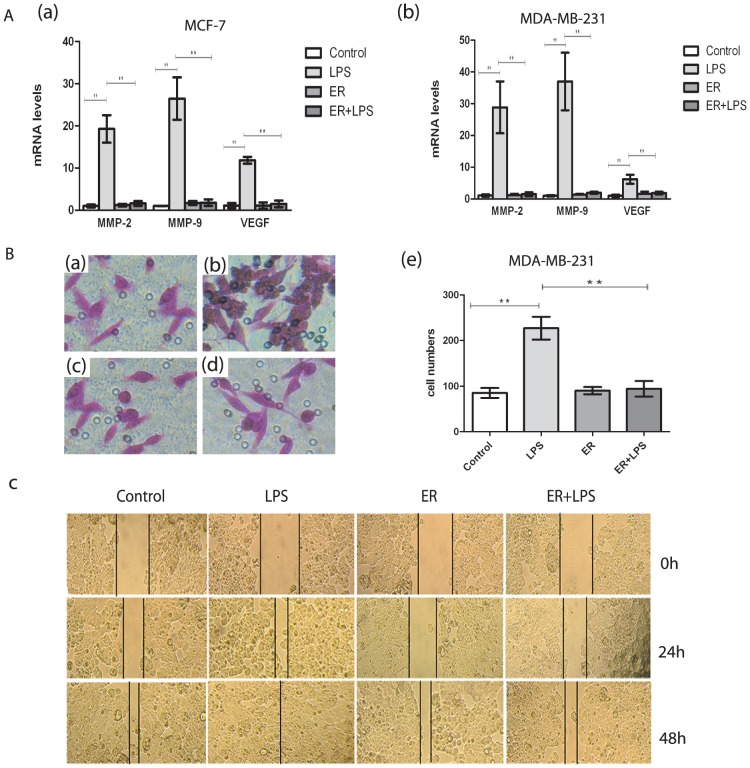
TLR4 antagonist blocked invasiveness and migration in human breast cancer cells. (A) mRNA of MMP-2, MMP-9 and VEGF were analyzed by real-time PCR. Each bar represented triplicate analyses of mean±SD, **P<0.05. LPS (2 µg/ml, 48 h), ER(100 nmol/L eritoran,30 min pretreatment, 48 h), ER+LPS(100 nmol/L eritoran,30 min prior to 2 µg/ml LPS, 48 h).(B) Invasiveness of MDA-MB-231 cells was performed by transwell assay. (a)Control,(b)LPS,(c)ER,(d)ER+LPS. Magnification, ×200. (e) Total number of invading cells through the matrigel was counted under the microscope in the whole fields. Each bar represents the mean±SD of three wells counted. **P<0.05. Results are representative of three separate experiments. (C) Wound-healing assay was performed to observe the changes in MCF-7 cells invasion. Magnification, ×100. All results were representative of three separate experiments. (B),(C) LPS (10 µg/ml, 48 h), ER(100 nmol/L eritoran,30 min pretreatment, 48 h), ER+LPS(100 nmol/L eritoran,30 min prior to 10 µg/ml LPS, 48 h).

Real time PCR revealed that the expression of TLR4 on both human breast cancer cell lines was blocked by TLR4 antagonist which was added prior to the addition of LPS in ER+LPS group compared with LPS group (P<0.05)(Fig. 6A). The protein levels of TLR4 and MyD88 were also reduced in ER+LPS group (P<0.05)(Fig. 6B). Incubation with eritoran did not lead to significant changes in TLR4 and MyD88 compared with control group. (Fig. 6) MMP-2, MMP-9 and VEGF mRNA expression in both cells was blocked in ER+LPS group compared with LPS group (P<0.05)(Fig. 7A). TLR4 antagonist inhibited LPS-induced migration of MDA-MB-231 cells. The migration number of the cells in ER+LPS group was significantly reduced in transwell assay (P<0.05) (Fig. 7B). In the wound-healing assay MCF-7 cells in ER+LPS group was healed clearly slower than LPS group (P<0.05) (Fig. 7C). Compared with control group, eritoran treatment alone didn't result in obvious changes in invasion and migration as well.(Fig. 7)

### TLR4 was overexpressed in human breast cancer tissue and correlated with lymph node metastasis

**Figure 8:**
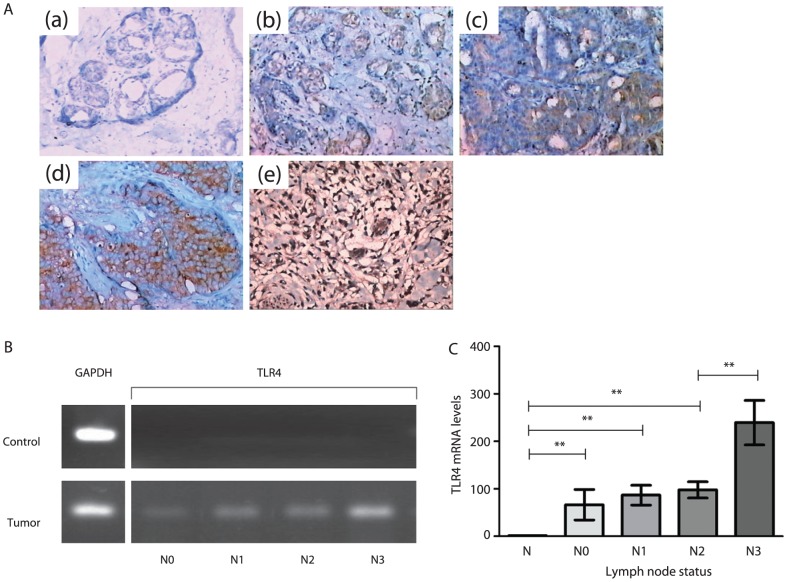
TLR4 expression in clinical tissue. (A) Immunohistochemistry for TLR4 in tissue sections. Magnification, ×200. (a) TLR4 expression in normal breast tissue. (b) TLR4 expression in lymph node metastasis (N0) breast cancer tissue. (c) TLR4 expression in lymph node metastasis (N1) breast cancer tissue. (d) TLR4 expression in lymph node metastasis (N2) breast cancer tissue. (e) TLR4 expression in lymph node metastasis (N3) breast cancer tissue. (B) mRNA of TLR4 in normal breast tissue and tumor tissue by RT-PCR. (C) mRNA of TLR4 in normal breast tissue and tumor tissue by real-time PCR. Each bar represents analyses of mean±SD, **P<0.05.

RT-PCR and immunohistochemistry showed that TLR4 was expressed in tumor tissue, however, normal breast tissue hardly expressed TLR4. In tumor tissue, TLR4 was localized in the cytoplasm and cell membrane, and its expression tended to be stronger in patients with lymph node metastasis of N0, N1, N2 and N3 (Fig. 8A). RT-PCR had similar results as shown in Fig. 8B. Using real-time PCR, we confirmed that tumor tissues expressed TLR4 more than normal breast tissues did. TLR4 mRNA expression in tumor tissues from N0 to N3 was 59.29±31.93, 77.43±20.72, 87.37±17.00 and 213.71±46.79 times higher, respectively, than in normal tissue (P<0.05). Although there was no significant difference among N0, N1 and N2 (P>0.05), TLR4 mRNA expression in tumor with N3 was significantly higher than the others (P<0.05), as shown in Fig. 8C.

## Discussion

The current study demonstrated that TLR4 expressed on human breast cancer cells may contribute to tumor progression, especially in metastasis. Our previous study identified that MDA-MB-231 cells expressed multiple TLRs, especially TLR4, and demonstrated that knockdown of TLR4 could actively inhibit proliferation and survival of breast cancer cells [17]. We focused our study on TLR4 and used the LPS ligand and antagonist eritoran to explore further the role of TLR4 in tumor metastasis. Compared with MCF-7 cells, MDA-MB-231 cells have higher invasive potential [19]. In our study, both cell lines were used to confirm more accurately the role of TLR4 in tumor metastasis.

Our results identified that TLR4 and MyD88 was expressed in MCF-7 and MDA-MB-231 human breast cancer cells, and the expression of TLR4 and MyD88 in both cell lines was increased notably is response to LPS stimulation and reduced by antagonist eritoran.

Invasion through basal membranes is a critical metastatic step for cell detachment from primary loci and intrusion into distant organs [20]. With an invasion assay, our findings indicated that TLR4 played an effective role in the invasive potential of MDA-MB-231 human breast cancer cells. However, MCF-7 cells barelyapp∶addword∶barely migrated through the transwells. The wound-healing assay was used to assess the impact of TLR4 stimulation with LPS on invasion of MCF-7 cells. The results showed that after LPS stimulation, the wound healed clearly faster than in the control group. Eritoran could block the invasiveness and migration of LPS induced MDA-MB-231 and MCF-7 cells.

Numerous clinical and experimental studies have demonstrated an increase in particular MMPs, especially MMP-2 and MMP-9 with cancer progression. Autocrine secretion of cytokines by cancer cells exerts an important effect in the control of cancer cell behavior such as adhesion and invasion [21], [22]. VEGF and MMP-9 are regarded as indispensable cytokines for breast cancer cell invasion and adhesion [23]. Moreover, our study indicated that, after TLR4 stimulation, breast cancer cells produced higher amounts of MMP-2, MMP-9 and VEGF, and as stimulus concentration increased within a certain range, the production of MMP-2, MMP-9 and VEGF also increased.

The link between systemic inflammation and promotion of tumor metastasis is well established [24]. Chronic infection and inflammation are considered two of the most prominent epigenetic and environmental factors contributing to oncogenesis and tumor progression [25]. IL-6 contributes to lung and breast cancer cell malignancy and effusion [26], [27], and IL-6 and IL-10 may bias the induction of the immune response and lead to a state of tolerance against tumors [28]. LPS-pretreated TLR4-positive/myeloid differentiation 88 (MyD88)-positive mantle cell lymphoma(MCL) inhibited the proliferation and cytolytic activity of T cells by secreted IL-10 and VEGF, and triggers a cascade that leads to MCL growth and evasion from immune surveillance [29]. Our results showed that LPS promotes MyD88 up-regulation in both cell lines, and MDA-MB-231 cells up-regulated the production of IL-6 and IL-10, in a dose-dependent manner of LPS, and MCF-7 up-regulated IL-6. We concluded that TLR4/MyD88 signaling contributed to their invasive activity of human breast cancer cells via autocrine and/or paracrine which played an active role in human breast cancer metastasis. However, in our study, IL-10 was not detected in MCF-7 supernatants which may be of its lower level or no production. This may be caused by the improper dose of LPS and treatment time which will be our follow-up research focus.

After establishing the significant effects of TLR4 stimulation by LPS in MDA-MB-231 and MCF-7 cells *in vitro*, we then investigated whether TLR4 stimulation also promoted tumorigenicity and metastasis in a mouse tumor model. *In vivo*, we showed that LPS stimulation increased TLR4 expression in MDA-MB-231 breast tumors in nude mice as determined by immunohistochemistry. Our data showed that higher TLR4 expression promoted tumorigenesis and metastasis. Tumor volume and weight in the LPS group were significantly higher than in the control group. The most direct evidence that higher TLR4 expression could promote metastasis was that metastatic lesions in the liver could be macroscopically as well as microscopically. Therefore, activating TLR4 from cancer cells could promote tumor metastasis.

Studies have shown the significance of TLR4 expression in pancreatic ductal adenocarcinoma [30] and its correlation with poor prognosis in colorectal cancer [31]. Herein, we explored a more direct relationship between TLR4 and human breast cancer. Tumor specimens confirmed that TLR4 was highly expressed in human breast cancer tissues as similar as others [32], [33] while normal breast tissue hardly expressed TLR4. Moreover, it is reported that TLR4 is expressed at low levels in normal human breast epithelium [34], [35]. This may caused by the individual variation of human samples which warrant further exploration in larger tissue samples in our later studies. More interestingly, we found that there was a tendency that expression of TLR4 in tumor tissues increased in association with lymph node metastasis. In particular, N3 tumor had significantly increased expression compared to patients with N0, N1 and N2, although there was no significant difference among N0, N1 and N2. These findings were direct and convincing evidence of the link between TLR4 and human breast cancer metastasis.

In conclusion, our data confirmed that human breast cancer cell lines and tissue expressed TLR4. The findings which human breast cancer cells showed significant biological changes after LPS stimulation *in vitro* and *in vivo*, and a tendency that TLR4 overexpression in tumor tissue was related with lymph node metastasis, support the concept that TLR4 played a significant role in breast cancer metastasis. Therefore, TLR4/MyD88 signaling molecules may be novel therapeutic targets in patients with breast cancer.

## Supporting Information

Table S1 **PCR primers for genes.** PCR primers for GAPDH, TLR4, MMP-2, MMP-9 and VEGF.
(DOC)

Table S2 **Patient characteristics.** Routine pathological parameters including age, tumor type, tumor grade, tumor stage, lymph node status, immunohistochemical determination of progesterone receptor and estrogen receptor status.
(DOC)
