# Supplementary material for: Toll-Like Receptor 4 Prompts Human Breast Cancer Cells Invasiveness via Lipopolysaccharide Stimulation and Is Overexpressed in Patients with Lymph Node Metastasis
Source: PLoS One. 2014 Oct 9;9(10):e109980. doi: 10.1371/journal.pone.0109980 (PMC4192367; doi:10.1371/journal.pone.0109980)
Supplement: Table S1 — PCR primers for genes. PCR primers for GAPDH, TLR4, MMP-2, MMP-9 and VEGF. (DOC) [file pone.0109980.s001.doc]

**Table S1 PCR primers** for genes

| Genes | Primer sequence（5’-3’） | Amplification size（bp） |
| --- | --- | --- |
| GAPDH | forword:GGATTTGGTCGTATTGGG  reverse:GGAAGATGGTGATGGGATT | 205 |
| TLR4 | forword:GAGCCGTTGGTGTATCTTTGA  reverse:CTCCCATTCCAGGTAGGTGTT | 166 |
| MMP-2 | forword:AGAGACAGTGGATGATGCCTTT  reverse: ATCGTCATCAAAATGGGAGTCT | 220 |
| MMP-9 | forword:GTGCTGGGCTGCTGCTTTGCTG  reverse: GTCGCCCTCAAAGGTTTGGAAT | 303 |
| VEGF | forword: GAGGAGCAGTTACGGTCTGT  reverse: GTAGCTCGTGCTGGTGTT CA | 371 |
