# Supplementary material for: Toll-Like Receptor 4 Prompts Human Breast Cancer Cells Invasiveness via Lipopolysaccharide Stimulation and Is Overexpressed in Patients with Lymph Node Metastasis
Source: PLoS One. 2014 Oct 9;9(10):e109980. doi: 10.1371/journal.pone.0109980 (PMC4192367; doi:10.1371/journal.pone.0109980)
Supplement: Table S2 — Patient characteristics. Routine pathological parameters including age, tumor type, tumor grade, tumor stage, lymph node status, immunohistochemical determination of progesterone receptor and estrogen receptor status. (DOC) [file pone.0109980.s002.doc]

**Table S2 Patient characteristics**

| **Number of malignant patients** | 22 |
| --- | --- |
| **Age**  Range  Median  **Histological type**  Ductal  **Histological grade**  Ⅰ/Ⅱ  Ⅲ  **Estrogen receptor status**  Positive  Negative  **progesterone receptor status**  Positive  Negative  **Ki-67 status**  Low (≤25%)  High (>25%)  **Tumor stage**  T1  T2  T3  **Lymph node status**  N0  N1  N2  N3 | 42-68  53.2  22  15  7  16  6  13  9  13  9  9  11  2  8  5  5  4 |
